# A Network Synthesis Model for Generating Protein Interaction Network Families

**DOI:** 10.1371/journal.pone.0041474

**Published:** 2012-08-13

**Authors:** Sayed Mohammad Ebrahim Sahraeian, Byung-Jun Yoon

**Affiliations:** Department of Electrical and Computer Engineering, Texas A&M University, College Station, Texas, United States of America; King's College London, United Kingdom

## Abstract

In this work, we introduce a novel network synthesis model that can generate families of evolutionarily related synthetic protein–protein interaction (PPI) networks. Given an ancestral network, the proposed model generates the network family according to a hypothetical phylogenetic tree, where the descendant networks are obtained through duplication and divergence of their ancestors, followed by network growth using network evolution models. We demonstrate that this network synthesis model can effectively create synthetic networks whose internal and cross-network properties closely resemble those of real PPI networks. The proposed model can serve as an effective framework for generating comprehensive benchmark datasets that can be used for reliable performance assessment of comparative network analysis algorithms. Using this model, we constructed a large-scale network alignment benchmark, called NAPAbench, and evaluated the performance of several representative network alignment algorithms. Our analysis clearly shows the relative performance of the leading network algorithms, with their respective advantages and disadvantages. The algorithm and source code of the network synthesis model and the network alignment benchmark NAPAbench are publicly available at http://www.ece.tamu.edu/bjyoon/NAPAbench/.

## Introduction

Protein-protein interactions (PPIs) lie at the core of a wide range of biological processes in cells, including transcriptional, signaling, and metabolic processes [1]. Recent technological advances have enabled the high-throughput measurement of these interactions in various species [2]–[4], and a variety of computational methods have been developed for in-silico prediction of protein interactions [5]–[8]. Availability of large-scale protein interaction data, typically represented as networks of interacting proteins, has opened up new ways for the systematic study of biological networks. Especially, cross-species comparison of genome-scale PPI networks can provide important insights into the structure and organization of biological networks, as well as important similarities and variations across different species [9]. In recent years, a large number of computational methods have been developed for comparative analysis of biological networks, where their main focus has been on the identification of functional modules that are conserved in the networks of multiple species [10]–[39]. These methods can be broadly divided into two categories, namely, network querying and network alignment. Network querying aims to identify subnetwork regions in the network of a target species that are similar to a small subnetwork of another species, used as query [32]–[39]. For example, this could be used for querying a known functional pathway in a well-studied species to identify putative homologous pathways in different species, thereby allowing knowledge transfer across species. Network alignment can be viewed as a generalization of network querying, and it aims to predict the best mapping between a set of networks, based on the similarity of the constituent molecules and their interaction patterns [10]–[31]. Network alignment methods may be used to analyze the cross-species variations of biological networks, to predict conserved functional modules, or to infer the function of unannotated proteins.

Research in comparative network analysis is still at an early stage, but many existing studies have demonstrated its potential as an effective tool for gaining important insights into biological systems, that would be otherwise difficult to obtain.

Unfortunately, further advance in comparative network analysis research is critically impeded by the lack of a gold standard for evaluating network alignment algorithms. Currently, there is no comprehensive and reliable benchmark dataset that can be used for validating these algorithms [12]. For this reason, it is common practice to assess the performance of network alignment algorithms in indirect ways, for instance, based on the functional coherence of the aligned nodes in the predicted network alignment or simply through anecdotal examples. Functional annotations based on Gene Ontology (GO) [40] or KEGG orthology (KO) [41] are often employed for this purpose. However, these annotations are mainly curated based on the sequence similarity between molecules, hence they may fail to effectively capture the actual functional coherence between the molecules [28], [42]. Considering that network alignment aims to incorporate molecular interaction data with sequence data to make predictions that are biologically more relevant, evaluating network alignment algorithms based on annotations that are strongly influenced by sequence similarity is certainly less than ideal. Besides, currently available protein interaction databases, such as BioGRID [43], MIPS [44], DIP [45], IntAct [46], MINT [47], and Human Protein Reference Database (HPRD) [48], include the protein interaction networks for only a few species, where the interaction data are very incomplete even for meta-databases – such as PINA [49] and APID [50] – that have been constructed by integrating multiple databases. For example, BioGRID v. 3.1.82 (November 2011), which is one of the most comprehensive among the existing PPI databases, contains the PPI networks of just 25 organisms, where the networks of 7 organisms – *A. thaliana*, *C. elegans*, *D. melanogaster*, *H. sapiens*, *M. musculus*, *S. cerevisiae*, and *S. pombe* – include more than few hundred interactions. It is widely suspected that a significant number of interactions in the current PPI networks may be spurious, while many true interactions may be still missing. As discussed in [51], based on the analysis of synthetic networks, incomplete knowledge poses a major challenge for interactome-level comparison between different species.

Considering the incompleteness of the current PPI networks, as well as the difficulty of accurately assessing the functional correspondence between proteins, a network synthesis model that can generate *families* of protein interaction networks with biologically realistic properties may provide a practical and effective alternative. Recently, Ali and Dean [51] have performed a simulation-based study, where a pair of evolutionary related synthetic networks were analyzed to investigate the source of low level of interaction conservation in network alignment results. Erten *et al.* [52] also proposed a simulation scheme for generating a set of networks with known phylogeny, where the driving motivation was to evaluate the accuracy of their network-based phylogeny reconstruction algorithm. These studies [51], [52] serve as interesting showcases of the important role of synthetic network models. However, these models have also a number of practical limitations. For example, the model presented in [51] cannot be used to synthesize a network family with an arbitrary phylogeny. Furthermore, both models in [51] and [52] do not explicitly represent the functional correspondence between individual proteins across different networks, which is indispensable for evaluating the accuracy of network alignment algorithms.

In this paper, we present a general network synthesis model that can effectively address these issues. Following a pre-specified phylogenetic tree, the model can generate a family of evolutionarily related protein interaction networks, whose properties closely mimic those of real networks – in terms of both the *internal* properties of the individual networks as well as the *comparative* properties across networks – as will be shown in our analysis. By internal network properties, we refer to the local characteristics (such as the node degree and the clustering coefficient) and their distributions over each network, which are important in understanding the overall topology. On the other hand, by comparative or cross-network properties, we refer to the properties that can be estimated through network comparison (e.g., sequence similarity between proteins that belong to different networks) and reflect the similarity (or the lack thereof) between networks, which arise from their evolutionary relationship. To demonstrate the utility of the network synthesis model, we created a comprehensive network alignment benchmark based on the proposed model and carried out an extensive performance analysis of select state-of-the-art network alignment algorithms.

## Methods

### Network Growth Models

In this section, we briefly review existing network growth models that aim to computationally simulate the evolutionary growth of a single biological network. Recently, there has been significant interest in developing network growth models [53]–[70] that can capture the characteristics of real biological networks, including PPI networks. As pointed out in [71], PPI networks do not follow the Erdös-Rényi's model for random graphs. Instead, the structure of biological networks appears to be governed by a scale-free degree distribution, which is also the case for social networks. The scale-free model suggests that the probability that a given node will have a degree (i.e., number of edges) of 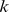 follows a power-law 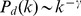, for some degree exponent 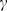. In general, a scale-free network possesses a few highly connected nodes (often referred as hubs), while the rest of the nodes have only a relatively small number of connections. This trend is generally observed in many PPI networks, which can be explained at a molecular level, at least in part, by the different degrees of protein binding specificity – i.e., the number of binding surfaces or binding partners – required by the cell for carrying out various biological functions [42]. *Preferential attachment (PA) growth model* [56] is one of the network evolution models that can generate such a distribution. In the PA model, the network is grown by iteratively adding a new node to the network and adding random connections to existing nodes. The probability of adding an edge to a given node is proportional to its degree, hence the model prefers to connect the new node to nodes that have many interacting partners. The PA model can also capture another important property of PPI networks called the “small-world effect”, which means that any node in the network can be typically reached from other nodes within a few links. Despite its effectiveness in modeling the scale-free degree distribution in PPI networks as well as their small-world property, the PA mechanism fails to capture other important properties, such as the graphlet distribution in real networks and their structural modularity [53], [65], [72], [73].

Inspired by the gene duplication model used to explain genome evolution [74], several duplication-based techniques have been proposed to simulate network evolution [53]–[55], [57]–[63], [66], [67], [69]. Basically, the gene duplication models assumes that the primary source of protein diversity is the repetitive duplication of existing genes followed by mutation of the duplicated genes leading to functional divergence [74]. A recent analysis of protein interaction networks [75] showed that gene duplication may play important roles in increasing the organismal complexity. The duplication-divergence model can generate networks that retain many of the generic characteristics of biological networks, such as the power-law degree distribution [76], hence it can provide an alternative framework for modeling PPI networks. The *duplication-mutation-complementation (DMC)* model [53] and the *duplication with random mutation (DMR)* model [54], [55] are two examples of duplication-divergence based network growth models that have been investigated in depth. Given a seed network, the DMC model [53] grows it by iterating the following steps:

1. Add a new node 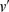 to the network by duplicating a randomly chosen node 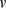 in the current network. Connect 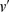 to all neighbors 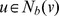 of the node 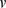.
2. For every neighbor 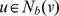, randomly pick either edge 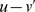 or 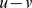, and randomly remove the edge with probability 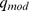.
3. Add a new edge between 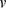 and 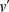 with probability 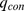.

It was shown that the above DMC model can capture various biological features of PPI networks [72], [77], including their hierarchical modularity. The DMR model is another well-studied network growth model based on the duplication-divergence principle [54], [55], where the network is obtained by repetitively applying the following steps:

1. As in the DMC model, add a new node 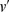 to the network by duplicating a randomly chosen node 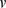 in the current network. Connect 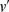 to all neighbors 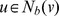 of the node 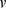.
2. Randomly remove the edges between 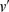 and 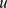 with probability 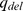.
3. Introduce random edges between 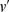 and other nodes in the network (that are not connected to the original node 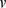) with probability 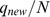, where 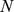 is the size of the current network.

As shown in [73], [78], the DMR model can generate networks that resemble real PPI networks in various aspects, such as the 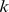-hop reachability (i.e, the number of distinct nodes that can be reached from a given node via a path of 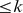 edges), the graphlet distribution, as well as the betweenness, closeness, and degree distributions.

Another notable network growth model that is not based on the duplication-divergence principle is the *crystal growth (CG)* model, recently proposed by Kim and Marcotte [65]. The CG model takes a highly module-oriented approach, which tries to emulate the physical process of growing protein crystals in solution. Kim and Marcotte [65] showed that the CG model can better explain many features of real PPI networks, including their network topology, their characteristic age distribution, and the spatial distribution of the subunits of different ages within protein complexes, hinting at a plausible physical mechanism of network evolution. Specifically, the capability to accurately capture age-dependent interaction patterns in PPI networks is an important advantage of the CG model, as this is one major drawback of existing models (e.g., duplication-based techniques). The CG model grows a seed network by iteratively adding new nodes as follows:

1. Define modules (i.e., dense local network regions) in the current network using Newman's algorithm [79]. Let 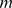 be the number of modules in the network.
2. Introduce a new node 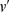 to the network. Either define the node 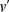 as a new module by itself (with probability 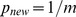) or add it to one of the existing modules (with probability 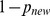).
3. If 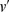 is defined as a new module, add 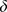 random connections to other nodes in the network according to the anti-preferential attachment (AP) rule. (Note that, according to the AP rule, nodes prefer to add edges to low-degree nodes.)
4. Otherwise, randomly select one of the 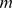 modules in the network and choose an anchor node 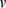 in the selected module, based on the AP rule. Add 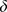 connections between 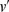 and the randomly selected neighbors of 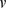. Repeat this step if 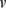 has less than 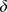 neighbors.

In addition to these three network growth models, there are also other randomized network generation schemes based on different approaches. For example, the scheme proposed in [70] does not generate a random network by growing a small seed network. Instead, this algorithm, which is developed based on Tailored random graphs, initiates from another random graph with the same dimensionality and the same degree sequence (i.e., the sequence of node degrees of the desired network) as the final network. Then it iteratively rewires the network (e.g., by edge swapping) to reach the desired degree distribution and joint degree statistics for connected nodes. However, this method is not well-suited for modeling network families, as it requires a predefined degree sequence (which may not be available in practice). Furthermore, as this scheme does not follow a growth model, it cannot effectively simulate evolutionarily related networks.

In the current work, we adopt and compare the three network growth models discussed above–i.e., DMC, DMR, and CG–to generate families of synthetic PPI networks. Note that the variables 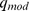, 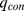, 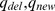 and 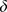 are user defined parameters for DMC, DMR, and CG schemes. Incorporation of other network evolution models is straightforward.

### Characteristics of Protein Interaction Networks

To develop a biologically realistic model for generating families of synthetic protein interaction networks, we first study the characteristics of real PPI networks of five organisms: *C. elegans*, *D. melanogaster*, *H. sapiens*, *M. musculus*, and *S. cerevisiae*. We present the analysis results for *D. melanogaster*, *H. sapiens*, and *S. cerevisiae*, which have the largest PPI networks among the five organisms, while the rest can be found in the supplementary data. The protein interaction data for these organisms have been obtained from IsoBase [80], a recently developed database of functionally related protein orthologs. IsoBase consists of the PPI networks of these five species, along with the homology scores between all pairs of proteins across different species, measured in terms of BLAST bit-value similarity of the protein sequences. The PPI networks in the IsoBase have been constructed by integrating the data in three different public databases: DIP [45], BioGRID [43], and HPRD [48]. Table 1 summarizes the statistics of IsoBase, which currently contains 48,120 proteins and 114,897 protein-protein interactions. From this table, we can also observe the incompleteness of the current PPI networks, evidenced by the large number of isolated proteins (i.e., proteins without known interactions). Furthermore, it also shows that only a small portion of the included proteins have known functional annotations according to the KEGG orthology. In the following, we investigate several important features that can be observed in these PPI networks.

**Table 1:** Statistics of the IsoBase database.

Species	*C. elegans*	*D. melanogaster*	*H. sapiens*	*M. musculus*	*S. cerevisiae*
# Proteins	19,756	14,098	22,369	24,855	6,659
# Interactions	5,853	26,726	43,757	452	38109
# Connected proteins	2,745	6,700	8,966	218	4,928
Average Degree	3.19	5.89	8.09	1.56	13.36
# Proteins with KO	2,102	3,366	4,195	3,805	1,605
# Connected proteins with KO	628	1,912	2,740	71	1,470
# Unique KO's	1,510	1,979	3,486	3,073	1,212

For each organism, the following numbers are shown: number of proteins in the network, number of interactions, number of connected proteins (those with interactions), average degree, number of proteins with KO annotations, number of connected proteins with KO annotations, and number of unique KO annotations in the network.

#### Intra-network properties of individual PPI networks

**Figure 1:**
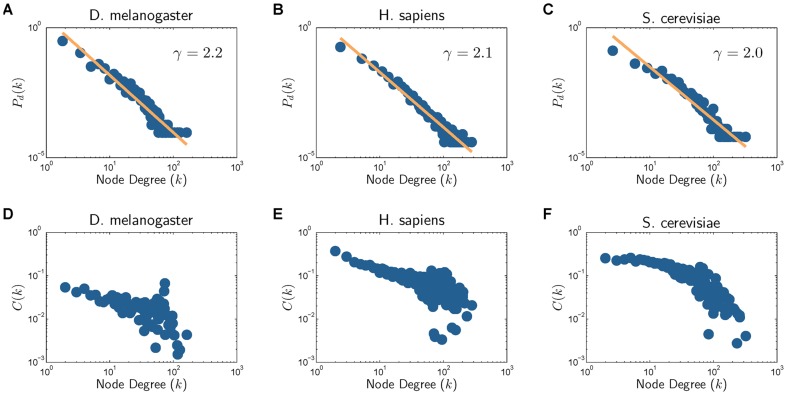
Network properties of various organisms. (A), (B), and (C) show the degree distributions, and (D), (E), (F) show the clustering coefficient profiles.

Two important network properties that we can typically observe in a real PPI network is the scale-free property and the modularity. The scale-free property manifests itself in the degree distribution 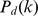, defined as the probability that a given node in the network will have 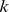 connections to other nodes, that follows a power-law distribution: 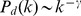 for some 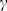. One measure that can be used to evaluate the modularity of a network is the clustering coefficient function 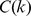. We define the clustering coefficient of a node 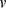 of degree 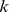 as 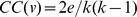, where 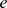 is the number of connections among the neighbors of 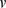. The clustering function 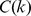 is defined as the average clustering coefficient of all nodes with 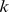 neighbors, and it is expected to scale down with 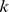 in a modular network. Figures 1(A)–1(F) and Figures S1(A)–S1(D) show the degree distribution 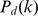 and the clustering coefficient function 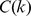 for the five organisms. These figures show that the degree distribution of each organism clearly follows a power-law distribution 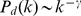, where 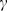 ranges between 1.8 and 2.3. We can also see that the clustering coefficient 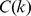 quickly scales down with 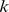 for all organisms, indicating the hierarchical modularity present in the PPI networks [71], [81].

#### Cross-network properties between different PPI networks

In order to devise a practical model for synthesizing a family of related networks, instead of a single network, it is important to investigate the cross-network properties that can be observed when comparing the PPI networks of different organisms. As discussed earlier, two aspects that are important in the comparative analysis of PPI networks are the *structural similarity* of the networks and the *molecular similarity* between the proteins that belong to different networks. The molecular similarity between proteins and their potential orthology is typically assessed based on their sequence similarity using a sequence alignment algorithm, such as BLAST [82] or FASTA [83]. Two questions of practical interest are: (i) how many potential orthologs would exist in different networks, for a specific protein in a given network, and (ii) how the protein similarity scores are distributed when comparing a network pair.

#### Distribution of potential orthologs

**Figure 2:**
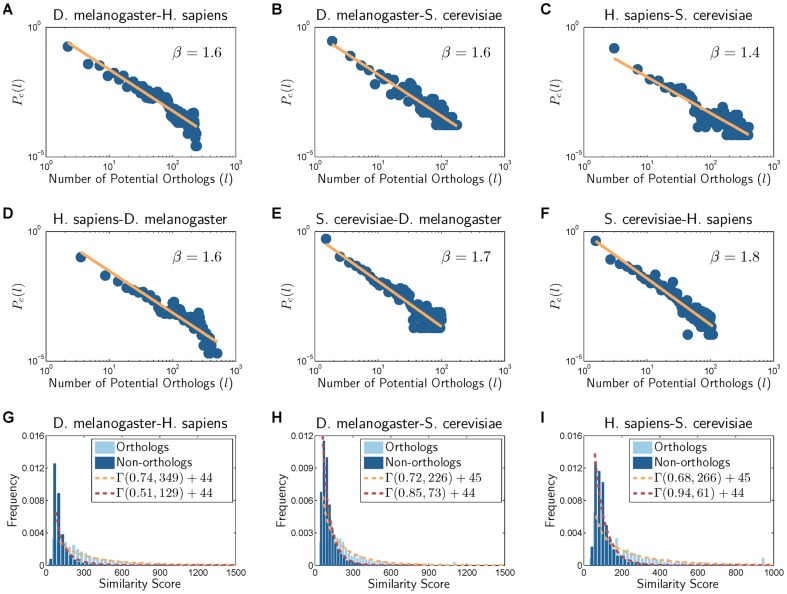
Cross-species network properties for different pairs of organisms. (A)–(F) show how the number of potential orthologs (i.e., nodes with high sequence similarity) are distributed between a given pair of networks. 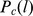 is the fraction of nodes with 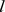 potential orthologs in the other network. (G)–(I) illustrate the sequence similarity (BLAST bit score) distribution for orthologous and non-orthologous node pairs.

Let 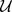 be the set of nodes (i.e., protein) in a PPI network 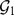 and 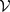 be the set of nodes in 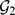. For a given node 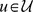 in the network 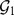, how many *potential orthologs* exist in the network 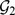
? By potential orthologs, we refer to pairs of proteins (in different PPI networks) that are candidates for being true orthologs according to their sequence similarity. Sequence similarity is often used as practical evidence for predicting protein orthology, and we assume that nodes with relatively high sequence similarity are more likely to be orthologous. Thus, we estimate the number of potential orthologs of each node 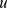 as
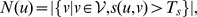
which is the number of nodes 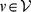 in the network 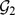 whose similarity score 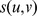 exceeds some threshold 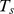. In practice, we may use a sequence alignment score, such as the BLAST bit score, to estimate 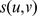. For any integer 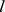, we define 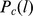 as the fraction of nodes 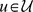 with 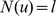. This relative frequency 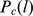 can provide useful insights regarding the presence of potential orthologs across different networks. Figures 2(A)–2(F) and Figures S2(A)–S2(N) show 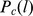 across all pairs of the five organisms in IsoBase, where a threshold of 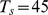 was used in all experiments. As shown in these figures, potential orthologs are generally sparse across networks. The results in Figure 2 and Figure S2 clearly reveal that the distribution 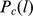 closely follows a power-law distribution 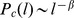 with an exponent 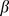 that ranges between 1.4 and 2.1. For example, let us consider the number of proteins in the *D. melanogaster* network that are potentially orthologous to proteins in the *S. cerevisiae* network. Among the 6,659 proteins in the *S. cerevisiae* network, 3,369 proteins do not have any potential orthologs in *D. melanogaster* whose sequence similarity score exceeds the threshold 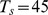. Among the rest, 1,707 proteins have no more than two potential orthologs in the *D. melanogaster* PPI network, 578 proteins have 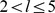 potential orthologs, 291 proteins have 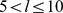 potential orthologs, 246 proteins have 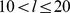 potential orthologs, 295 proteins have  potential orthologs, 130 proteins have 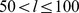 potential orthologs, and only 43 proteins have more than 100 potential orthologs. The general trend does not significantly change for choosing a different threshold 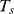. For example, even when we raise the threshold to 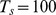, the number of proteins in *S. cerevisiae* with more than 50 potential orthologs in *D. melanogaster* would just decrease to 33. The results are similar for other network pairs, which show that there are typically only a few nodes in a PPI network with a relatively large number of potential orthologs, while most nodes only have a small number of potential orthologs, if any, in other organisms. This observation reveals an important challenge in network alignment, namely, strong reliance on sequence similarity can lead to predictions that are biologically insignificant and misleading, and effective incorporation of interaction data is crucial to minimize this risk.

#### Distribution of sequence similarity scores

Now, let us consider the distribution of the similarity score between nodes in different networks. As before, let 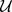 be the set of nodes in a PPI network 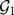 and let 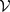 be the set of nodes in a different PPI network 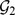. We define the set of orthologous proteins in the two networks as
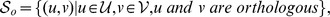
and the set of non-orthologous proteins as
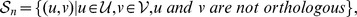
where 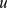 (in network 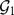) and 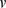 (in 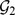) are regarded as orthologs if they belong to the same KEGG ortholog group, thus share the same functional annotation. We define 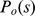 as the distribution of the similarity score 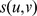 for orthologous nodes 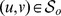. Similarly, we define 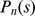 as the score distribution for non-orthologous node pairs 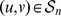. These distributions are shown in Figures 2(G)–2(I) and Figures S3(A)–S3(G) across all pairs of the considered organisms. These results show that the score distribution can be closely approximated by the Gamma distribution 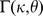, whose probability density function 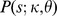 is defined as follows
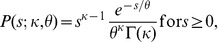
(1)for some shape parameter 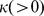 and scale parameter 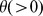. These figures also show that there is a substantial overlap between 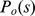 and 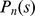, the similarity score distribution for orthologs and that for non-orhologs, which again reveals the the importance of incorporating interaction data into comparative networks analysis. This observation also confirms the results in previous studies [28], [42], [67], which showed that proteins that are conserved at the sequence level may fail to have conserved functionalities at the network level.

### Proposed Network Synthesis Model

Following the previous discussions, in this section, we propose a novel network synthesis model that can generate a family of evolutionarily related protein-protein interaction networks. Suppose we want to generate a family of 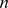 synthetic PPI networks 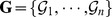. Each network 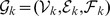 consists of set 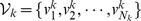 of 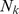 nodes; a set 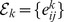 of 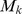 edges, where 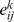 denotes the edge between node 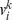 and 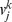; and a set 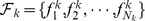, which maps each node 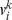 to a functional group 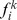 in 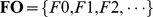, the set of all functional orthology (FO) annotations. A node 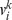 with 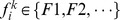 is regarded as an annotated protein with a known function 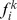, while it is regarded as an unannotated protein if 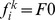. We define 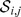 as a 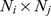 similarity score matrix that contains the sequence similarity score between all pairs of proteins for the networks 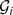 and 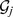. The set 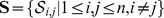 consists of the scoring matrices for all pairs of networks.

To generate the 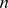 networks, we first specify the hypothetic phylogenetic tree 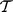 that describes the evolutionary relationship among the networks. The tree 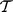, which is assumed to be a binary tree, will have exactly 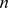 leaf nodes, in addition to a number of internal nodes, which correspond to the 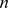 networks to be generated by the model. The basic idea of the proposed method is to follow the phylogenetic tree 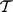 to create a set of related networks through repetitive network duplication, mutation, and network extension, starting from a single hypothetical ancestral network 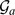. In order to create a biologically realistic ancestral network 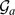, we begin by generating a small *seed network* and iteratively extend it using one of the network growth models – DMC, DMR, and CG models – described earlier. As discussed in [73], choosing the right seed network is crucial to capture the key topological features of real PPI networks. For the duplication-based models (i.e., DMC and DMR), we use a seed network that is similar to the one presented in [73], which was shown to accurately characterize the attributes of the *S. cerevisiae* PPI network. This seed network of size 50 includes two cliques (complete subgraphs), one with 10 nodes and the other with 7 nodes. Nodes in each of these two cliques are randomly connected to a few nodes in the other clique. The other 33 nodes are randomly connected to one of the 17 clique nodes. The nodes in the first and the second cliques are assigned to distinct functional groups 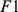 and 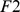, respectively. Each of the remaining 33 nodes is assigned to a different functional group, from 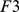 to 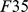. For the CG model, we use a seed graph of size 4 as in [65]. The initial seed network is grown into the ancestral network 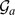 of size 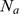 by employing one of the network extension models. While growing the network, every new node is assigned to a new functional group of its own.

**Figure 3:**
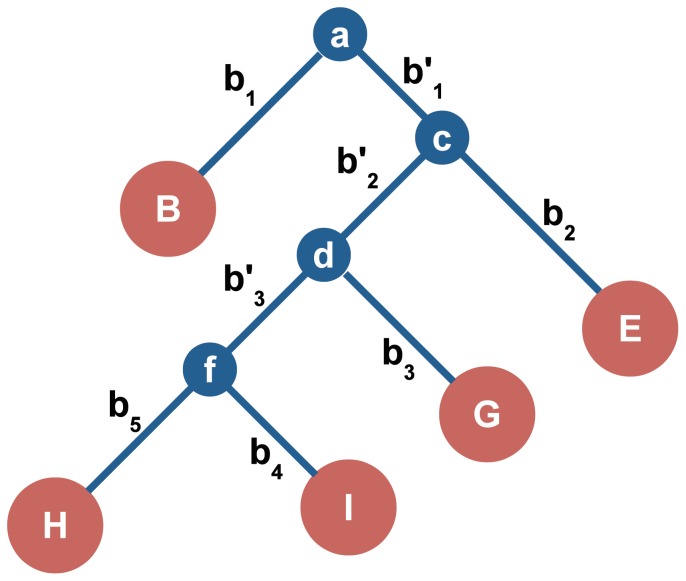
The phylogenetic tree of five hypothetical organisms.

Once the ancestral PPI network 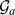 is created, we traverse the phylogenetic tree 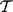 to generate descendant networks that are evolutionarily related to 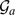. Figure 3 illustrates an example of a phylogenetic tree 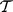 for five hypothetical species, which correspond to the five leaf nodes 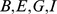, and 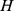. The tree also includes three internal nodes 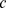, 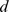 and 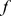, and the root node 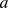. Since the phylogenetic tree is assumed to be binary, each internal node (including the root node) branches off to two child nodes. For each child node, we create a network by duplicating the parent network and evolving it into a larger network. For example, according to the tree in Figure 3, we generate two networks 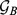 (for the leaf node 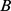) and 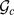 (for the internal node 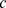) based on the ancestral network 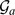 that corresponds to the root node 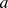, which is the parent of 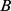 and 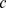. We will traverse the tree 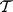 through a breadth-first search [84] and repeat this bifurcation process until all 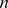 networks are generated. It is straightforward to see that this will require 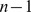 bifurcations, in total.

The bifurcation step is carried out as follows. Suppose 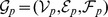 is the network that corresponds to the current internal node. We denote 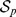 as the set of scoring matrices that contain the similarity scores between proteins in 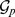 and those in the networks for other nodes in 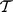 that have been previously visited. We generate the networks 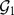 and 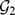 for the two child nodes by duplicating the parent network: 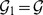 and 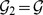. Both networks inherit the functional annotations of their parent 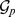 and the set 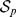 of scoring matrices. For every pair of nodes 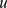 in 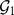 and 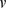 in 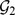, we randomly assign their similarity score according to a Gamma distribution as follows:
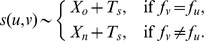
(2)where 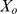 and 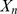 are random numbers sampled according to 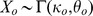 and 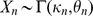. Note that the similarity score 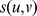 takes a different distribution, depending on whether or not 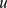 and 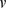 have the same functional annotation: 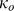 and 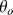 are the shape and scale parameters of the Gamma distribution for orthologs (with identical FO annotations); 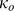 and 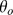 are the parameters for non-orthologs (with different FO annotations). 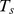 is used to simulate the thresholding effect of sequence similarity scores. As we have seen in our analysis of real PPI networks, potential orthologs across different networks are generally sparse. In the proposed model, we enforce the number of potential orthologs to follow a power-law distribution 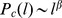, as in real PPI networks.

To diverge the child networks 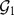 and 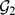 from the parent network 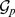, we independently apply a network growth algorithm (DMC, DMR, or CG) to each of these networks. In this step, the number of new nodes added to each child network may be specified according to the evolutionary distance between the corresponding hypothetical species in the tree 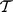. For instance, in Figure 3, the number of additional nodes (referred as the “length” of a given branch) are shown along the branches. In this example, if the ancestral network has 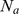 nodes, the PPI network 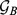 for node 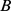 will have 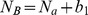 nodes and the PPI network 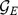 for node 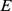 will have 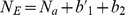 nodes. Consider a new node 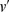 that was either (i) obtained by duplicating an existing node 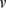 (when using either the DMC or the DMR model) or (ii) a new node whose anchor node was chosen to be 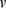 (when using the CG model). We transfer the functional annotation and the similarity scores from an existing node 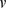 to a new node 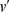 as follows:

1. With probability 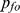, assign 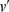 to the same functional group as 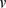 by setting 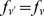. With probability 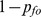, set 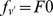, which implies that 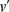 takes a new unknown function.
2. For every protein 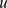 in the networks that correspond to previously visited nodes in 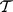, assign the similarity score between 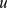 and 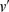 as:
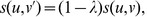
(3)where 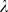 is a random scaling factor with a uniform distribution over 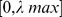. The upper bound 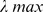 (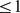) specifies the extent of the sequence-level divergence between 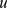 and 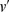.

In this way, we can model the functional inheritance and the sequence similarity between the duplicated nodes, where a duplicated node may have a different function from the original node. Finally, when using the CG model, a new node 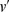 that forms a new functional module by itself, hence not anchored to any of the existing nodes, will be assigned a new unannotated function (i.e., 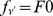).

## Results and Discussion

### Attributes of Synthetic Networks

To validate the proposed network synthesis model, we generated synthetic PPI networks according to the model and analyzed the individual and cross-species characteristics of the synthesized networks. We first generated an ancestral network 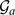 of size 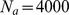. A simple binary tree with two leaves was used to evolve 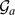 into two networks 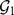 and 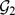, respectively with 5,000 nodes and 7,000 nodes. For network extension, we applied all three network growth models – DMC, DMR, and CG – discussed in this paper. For DMC, we used 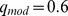 and 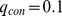 as in [65]. For DMR, we set the parameters to 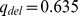 and 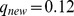 as in [73]. We used 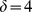 for CG as in [65]. The scaling and shape parameters of the Gamma distributions in (2) were set to 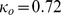, 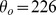, 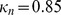, 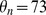, and the exponent 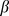 in the distribution 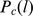 was set to 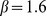, such that the cross-network properties between 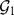 and 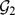 resemble those between the *D. melanogaster* PPI network and the *S. cerevisiae* PPI network. The parameters 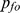 and 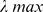 that control the functional inheritance and sequence similarity between orthologous nodes were set to 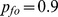 and 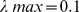, so that protein function and sequence similarity is conserved at the 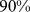 level. Although it is practically difficult to accurately determine these two parameters in real networks, the analysis in [85] shows this rate of functional conservation for duplicated genes.

In the case of CG algorithm, we made a slight modification in the first step of the algorithm as follows. In the original algorithm proposed in [65], when adding a new node, the modules of the current network are recomputed at each iteration. To speed up the CG algorithm, we instead redefine the modules every 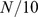 steps, where 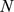 is the size of the current network. In other words, in the early iterations, we redefine modules in every iteration, while as the network grows larger, we apply the module redefinition step only occasionally and use these modules over multiple iterations. Simulation results show that the CG method can still accurately capture the generic features of real PPI networks with this modification. We leave the module redefinition frequency as a control parameter that can be freely adjusted.

**Figure 4:**
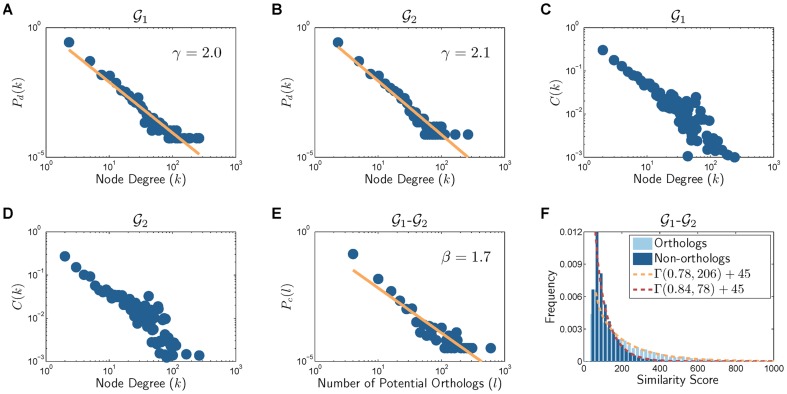
Properties of the networks generated using the DMC model. (A)–(B) Degree distribution. (C)–(D) Clustering coefficient profile. (E) Distribution of the number of potential orthologs. (F) Sequence similarity distribution for orthologous nodes and the distribution for non-orthologous nodes. (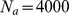, 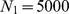, 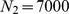, 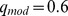, and 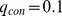).

**Figure 5:**
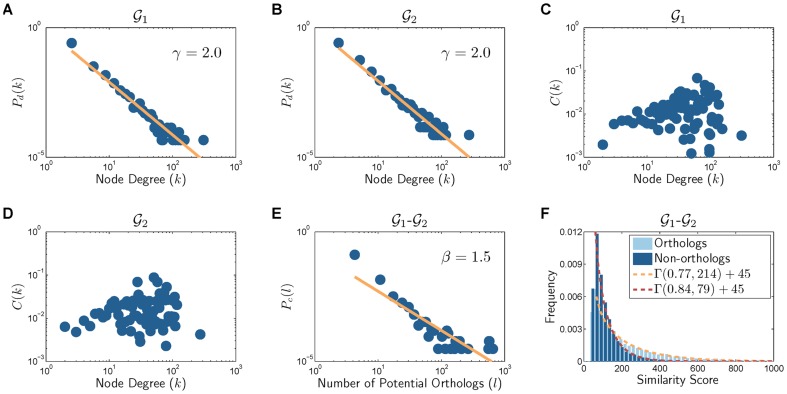
Properties of the networks generated using the DMR model. (A)–(B) Degree distribution. (C)–(D) Clustering coefficient profile. (E) Distribution of the number of potential orthologs. (F) Sequence similarity distribution for orthologous nodes and the distribution for non-orthologous nodes. (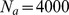, 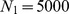, 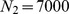, 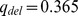, and 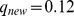).

**Figure 6:**
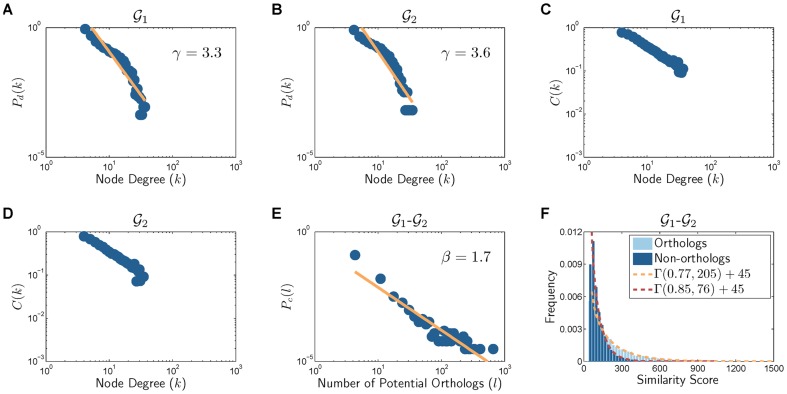
Properties of the networks generated using the CG model. (A)–(B) Degree distribution. (C)–(D) Clustering coefficient profile. (E) Distribution of the number of potential orthologs. (F) Sequence similarity distribution for orthologous nodes and the distribution for non-orthologous nodes. (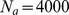, 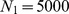, 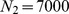, and 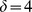).

The properties of the synthetic PPI network are shown in Figure 4, Figure 5, and Figure 6, for using DMC, DMR, and CG, respectively. As can be seen in these figures, all three schemes can accurately model the scale-free degree distribution. However, it appears that the hierarchical modularity can be better captured by using either DMC or CG, rather than DMR. Regarding the cross-network properties, these results also clearly show that the proposed network synthesis model can effectively capture the attributes of real PPI networks. For example, this can be immediately seen by comparing the network properties of 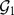 and 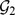 in Figures 4(E) and 4(F) (when using DMC) with those of the *D. melanogaster* and the *S. cerevisiae* PPI networks shown in Figures 2(B) and 2(H). Similar observations can be made from Figures 5(E) and 5(F) (for DMR) as well as Figures 6(E) and 6(F) (for CG).

### Construction of Network Alignment Benchmark

The network synthesis model presented in this paper provides an effective framework for generating network families with diverse characteristics. Such network sets may be used to assess the performance of various alignment techniques to identify their respective strengths and weaknesses under different conditions and problems settings. Furthermore, the proposed network synthesis model may be potentially used to expose previously unknown biases that a network alignment technique may have towards specific types of networks, thereby leading to better alignment techniques.

To demonstrate the utility of the proposed network generation scheme, we used it to create synthetic benchmark datasets that can be used for evaluating and comparing the performance of various network alignment algorithms. We call the proposed **N**etwork **A**lignment **P**erformance **A**ssessment **bench**mark as NAPAbench. In total, we generated three suites of datasets. The first suite (referred as the *pairwise alignment* dataset) contains three pairs of networks, where the respective network pairs were generated using DMC, DMR, and CG, respectively. Each pair consists of a network 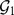 with 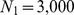 nodes and another network 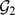 with 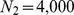 nodes, both evolved from an ancestral network 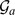 with 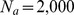 nodes, following a binary tree with two leaves. The second suite (referred as the *5-way alignment* dataset) contains three network families, each with five networks generated using DMC, DMR, or CG. To generate the network family, we first created an ancestral network 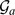 with 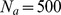 nodes. The phylogenetic tree 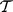 in Figure 3 was used to evolve 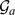 into five networks – 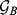, 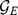, 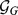, 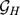, and 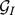 – which correspond to the five leaf nodes. For every branch, we set its length to 500. Thus, the size of the five networks were 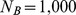, 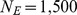, 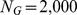, 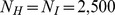. This dataset simulates a family of PPI networks that correspond to distantly related species. Finally, the third suite (referred as the *8-way alignment* dataset) also consists of three network families, each with eight networks generated by one of the three network extension models. The eight networks were obtained by evolving an ancestral network 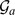 of size 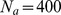 according to a full binary tree with eight leaf nodes. The branch length was set to 200 for all branches, which gave rise to eight equally sized networks, each with 1,000 nodes. This 8-way alignment dataset tries to simulate a network family of closely-related species. All the datasets in NAPAbench are publicly available at http://www.ece.tamu.edu/bjyoon/NAPAbench/.

### Performance Analysis of Network Alignment Algorithms

The created benchmark datasets, NAPAbench, can be used for reliable and comprehensive performance evaluation of existing network alignments. In this work, we used this synthetic benchmark to assess the performance of five well-known multiple network alignment algorithms: IsoRank [13], IsoRankN [12], NetworkBLAST-M [15], Græ mlin 2.0 [11], and MI-GRAAL [25]. *IsoRank* [13] uses spectral graph theory to evaluate the overall similarity between nodes that belong to different networks. This pairwise alignment score is computed for every node pair across all pairs of networks, which is then used to build the multiple network alignment according to a greedy approach. *IsoRankN* [12] further extends the idea in IsoRank by employing a spectral clustering scheme based on the pairwise node alignment scores. *NetworkBLAST-M* [15] computes the network alignment by first constructing a layered alignment graph based on the potential orthologous nodes, and then greedily searching for highly conserved local regions in the alignment graph. *Græ mlin 2.0* [11] takes a progressive approach to construct a global alignment of multiple networks, where it repeatedly performs pairwise network alignments according to a given phylogenetic tree that describes the relationship among the networks. The alignment is predicted by maximizing an objective function based on parameters that are learned from a set of known alignments. Finally, *MI-GRAAL* [25] is a recently proposed pairwise network alignment scheme that can integrate any number and type of similarity measures between network nodes, such as sequence similarity, structural similarity, and topological similarity.

Recall that the node similarity score in the proposed model tries to mimic the BLAST bit scores. Since NetworkBLAST-M and MI-GRAAL employ the BLAST E-values, instead of the BLAST bit scores, we should transform the bit scores into the corresponding E-values for these two algorithms. As discussed in [86], [87], the simulated bit score (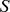) is related to the E-value (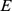) as 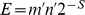, where 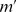 is the length of the BLAST query and 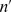 is the length of the target sequence. Here, we transform our simulated bit scores to E-values using 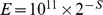 (assuming, for instance, the case when we BLAST a protein sequence with 500 residues in a database that contains a total of 200,000,000 residues). In this paper, we used the restricted-order version of NetworkBLAST-M as the running time of the relaxed-order version increases exponentially with respect to the number of networks to be aligned. As Græ mlin needs to learn the parameters of its scoring function in advance, we generated a training set that consists of five networks (with 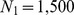, 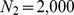, 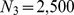, 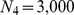, and 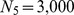 nodes, respectively), using the proposed scheme with the DMC model by following the tree shown in Figure 3. MI-GRAAL can integrate different kinds of similarity measures into the search process. Here, we adopt the graphlet degree signature distance and the E-values (measuring the sequence similarity) for MI-GRAAL alignment algorithm. For IsoRank and IsoRankN, the parameter 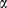, which determines the balance between sequence similarity and topological similarity, was set to 0.6.

The accuracy of each network alignment algorithm was assessed using four measures – specificity, correct nodes, mean normalized entropy, and coverage – which had been previously used in [11] and [12]. We refer the set of aligned nodes (i.e., potential orthologs) as the *equivalence class*. Each equivalence class may include an arbitrary number of nodes from each species. To compute the accuracy measures, we first removed the unannotated nodes from the alignment (i.e, nodes with the annotation 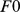) and then removed equivalence classes containing only a single node. A given equivalence class is viewed as being *correct* if all the included nodes belong to the same FO group. The four measures are defined as follows:

- **Specificity (SPE)**: The relative number of correctly predicted equivalence classes.
- **Correct Nodes (CN)**: The total number of nodes (i.e., proteins) that are assigned to the correct equivalence class. This measure reflects the sensitivity of the prediction [11].
- **Mean normalized entropy (MNE)**: The mean normalized entropy of the predicted equivalence classes can provide an effective measure of the consistency of the predicted network alignment. The normalized entropy of a given equivalence class 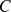 is computed as:
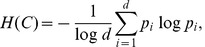
(4)where 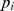 is the fraction of proteins in 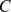 with the FO annotation 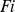, and 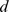 is the number of different FO groups. Thus, a cluster that consists of nodes with higher functional consistency will have lower entropy.
- **Coverage**: For any integer 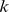, the total number of equivalence classes that contain nodes from 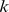 species. We report this measure only for multiple network alignment experiments (and not for pairwise alignments).

NetworkBLAST-M reports only the local alignment of the input networks, while the other four algorithms yield the global alignment of the given networks. For a fair comparison between these algorithms, we first convert the local alignment predicted by NetworkBLAST-M into a global network alignment by merging all local node correspondences. For example, if nodes 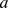 and 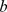 are aligned in one local alignment while 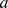 and 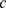 are aligned in another local alignment, we assume that 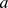, 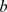, and 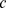 belong to the same equivalence class.

**Figure 7:**
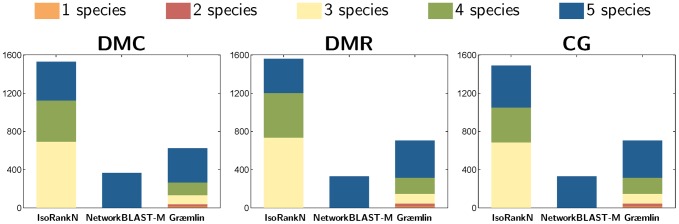
Number of equivalence classes in the 5-way alignment experiment that contain nodes from 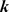
 **species (** **
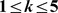
**).****

**Figure 8:**
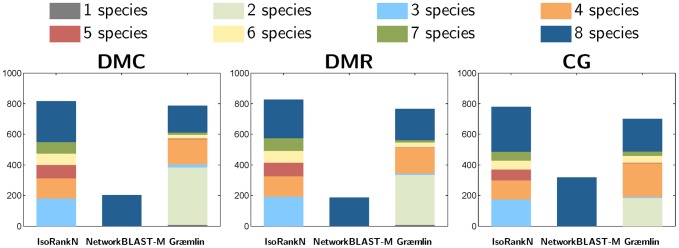
Number of equivalence classes in the 8-way alignment experiment that contain nodes from 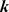
 **species (** 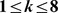 **).**

The SPE, CN, and MNE of the five algorithms are summarized in Table 2, Table 3, and Table 4, for the pairwise alignment dataset, 5-way alignment dataset, and the 8-way alignment dataset, respectively. Figure 7 and Figure 8 shows the coverage of different algorithms for the 5-way and 8-way dataset, respectively.

**Table 2:** Performance of different alignment algorithms on the pairwise alignment dataset of NAPAbench.

	DMC			DMR			CG		
	SPE	CN	MNE	SPE	CN	MNE	SPE	CN	MNE
IsoRank	77.53	**3883**	24.29	77.77	3914	23.92	77.22	3986	24.47
IsoRankN	82.69	3836	14.13	83.55	3915	13.40	83.16	3868	13.34
NetworkBLAST-M	**96.34**	3354	**5.33**	**96.60**	3005	**4.28**	**95.86**	**4646**	**4.44**
Græ mlin	77.37	2137	15.70	81.03	2322	13.33	90.72	2549	7.96
MI-GRAAL	66.13	3612	35.27	69.97	3852	31.59	79.48	4385	22.76

Performance comparison based on the pairwise alignment of two networks of size 3,000 and 4,000. The performance of each method is assessed using the following metrics: specificity(SP), number of correct nodes (CN), and mean normalized entropy (MNE). In each column, best performance is shown in bold.

**Table 3:** Performance Comparison on the 5-way network alignment dataset of NAPAbench.

	DMC			DMR			CG		
	SPE	CN	MNE	SPE	CN	MNE	SPE	CN	MNE
IsoRankN	**80.91**	**5538**	10.27	**79.58**	**5496**	11.14	**82.68**	**5689**	9.72
NetworkBLAST-M	62.18	1774	12.72	67.66	1591	10.62	69.90	3225	9.31
Græ mlin	51.07	3028	16.32	50.88	3100	16.94	62.89	4451	13.19
IsoRankN (only 5-species)	69.67	1859	**9.67**	68.07	1610	**10.26**	73.83	2223	**7.99**
Græ mlin (only 5-species)	35.90	1575	19.50	36.60	1581	20.29	54.44	2394	14.17

Performance comparison based on the 5-way alignment of five networks of size 1500, 2000, 2500, 3000 and 3000. The last two rows are obtained by considering only equivalence classes that contain at least one node from every species. The performance of each method is assessed using the following metrics: specificity(SP), number of correct nodes (CN), and mean normalized entropy (MNE). In each metrics, best performance is shown in bold.

**Table 4:** Performance Comparison on 8-way network alignment dataset of NAPAbench.

	DMC			DMR			CG		
	SPE	CN	MNE	SPE	CN	MNE	SPE	CN	MNE
IsoRankN	**64.50**	**4069**	13.62	62.52	**3938**	14.58	61.18	**3890**	14.58
NetworkBLAST-M	54.06	1166	13.97	**63.72**	1203	**10.65**	**63.66**	2236	10.84
Græ mlin	58.67	2315	16.51	51.34	1939	19.38	49.29	2729	17.24
IsoRankN (only 8-species)	56.74	1987	**10.06**	54.36	1797	10.81	54.30	2172	**10.33**
Græ mlin (only 8-species)	13.08	345	29.83	9.87	291	31.63	25.66	802	20.78

Performance comparison based on the 8-way alignment of eight networks of equal size 1,000. The last two rows are obtained by considering only equivalence classes that contain at least one node from every species. The performance of each method is assessed using the following metrics: specificity(SP), number of correct nodes (CN), and mean normalized entropy (MNE). In each column, best performance is shown in bold.

For pairwise network alignments, NetworkBLAST-M boasts significantly higher specificity and consistency (reflected in lower MNE) compared to other algorithms. IsoRank, IsoRankN, and MI-GRAAL yielded the highest number of correctly aligned nodes (i.e., CN) for networks generated using the DMC/DMR growth models, implying high sensitivity. For the networks created using the CG model, which yield highly modular networks, NetworkBLAST-M showed highest sensitivity, closely followed by MI-GRAAL.

For the 5-way and 8-way alignment experiments, we can clearly observe the degradation in sensitivity of NetworkBLAST-M, as shown in Table 3 and Table 4. This may be due to the fact that NetworkBLAST-M aims to predict equivalence classes that are conserved across all the compared species, as illustrated in Figure 7 and Figure 8. In these experiments, Græ mlin showed moderate performance, where the sensitivity was higher than NetworkBLAST-M, but the specificity and the consistency were lower. The multiple network alignment experiments based on the 5-way and the 8-way benchmark datasets in NAPAbench show that IsoRankN can yield the most accurate network alignment results, in terms of specificity, sensitivity, and consistency. This observation is in agreement with the performance assessment in [12], based on five real biological networks.

To compared the performance of different algorithms in predicting equivalence classes conserved across all networks, we also estimated the accuracy of IsoRankN and Græ mlin only for such classes. These results are shown in the last two rows of Table 3 and Table 4. We can see that IsoRankN still outperforms NetworkBLAST-M in most cases for 5-way alignment. In the 8-way network alignment, IsoRankN appears to outperform NetworkBLAST-M for networks generated using the DMC growth model. However, NetworkBLAST-M is more sensitive on networks obtained using the DMR model, and it is also more sensitive and more specific for networks generated using the CG model. These results also show that Græ mlin is outperformed by the other two algorithms in this case, which implies that it may not be effective in predicting orthologous nodes that are conserved across all species.

Figure 7 shows the number of equivalence classes (i.e., the coverage) that are predicted in the 5-way alignment dataset by the respective algorithms. In each case, the total number of equivalence classes is split into the number of classes that consist of nodes from 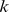 different networks (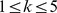). As shown in this figure, all three algorithms predicted similar number of equivalence classes that contain nodes from all 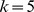 networks. However, we can see that IsoRankN predicts a significantly larger number of equivalence classes with 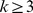 compared to the other algorithms. Considering that the 5-way alignment dataset consists of networks with varying size, equivalence classes that contain nodes from 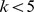 networks are fairly common, hence the ability of identifying such equivalence classes is certainly an important advantage of IsoRankN. Figure 8 shows coverage of different algorithms on the 8-way dataset. The trends are similar as in the 5-way alignment, and we can see that IsoRankN results in greater coverage for equivalence classes spanning 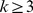 networks. Another interesting observation is that Græ mlin predicts a large number of equivalence classes that contain only nodes from 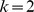 networks.

**Figure 9:**
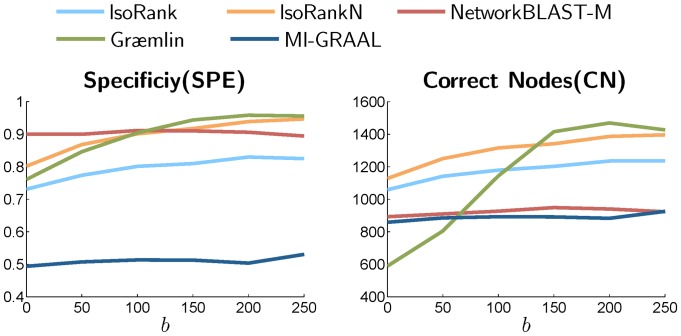
The specificity (SPE) and the CN (which reflects the sensitivity) of different alignment algorithms for varying level of separation between the similarity score distribution for orthologs and the score distribution for non-orthologs. Increasing the bias 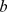 increases the separation between the two score distributions, hence increase the discriminative power of the node similarity score for predicting potential orthologs.

Next, we investigate the effect of sequence similarity on the performance of the various network alignment algorithms. To this aim, we add a bias term 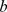 to the similarity score distribution of potential orthologs in (2), such that the score is randomly sampled as 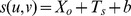, where 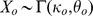. Increasing the bias 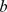 will further separate the similarity score distributions of orthologous and non-orthologous nodes. As a result, the larger 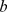 is, the easier it becomes to align the networks (and to predict the potential orthologs across networks) based on sequence similarity alone, without utilizing the topological similarity between networks. For this experiment, we generated two networks with 1,000 nodes from an ancestral network of size 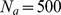. Figure 9 shows how specificity (SPE) and CN (which reflects sensitivity), change for varying values of 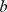 between 0 and 250. As can be seen in this figure, as the separation between the score distributions of orthologs and non-orthologs increases, both the specificity and the sensitivity are improved for IsoRank, IsoRankN, and Græ mlin. On the other hand, NetworkBLAST-M and MI-GRAAL display a constant level of accuracy that does not depend on the amount of separation. This implies that the first three alignment algorithms rely on the similarity between nodes relatively strongly when predicting the network alignment, while NetworkBLAST-M and MI-GRAAL use the similarity score mainly to predict potential orthology and do not rely too much on the extent of the similarity. In these experiments, Græ mlin appears to most strongly rely on the node similarity among the compared algorithms. In fact, Græ mlin achieves the highest specificity and sensitivity when there is a large separation between the score distributions (e.g., 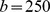), while resulting in the lowest sensitivity when the separation is small (e.g., 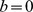).

Table 5 compares the computational complexity of the five algorithms, in terms of the total CPU time needed to align the networks in the respective datasets. All experiments have been performed on a desktop computer with a 2.2GHz Intel Core2Duo CPU and 4GB memory. It should be noted that Græ mlin requires a training stage for estimating the parameters used by the algorithm, which took more than a day in our experiments. The CPU time shown in Table 5 reveals that Græ mlin (without considering the training stage) and NetworkBLAST-M are the fastest among the five algorithms, while IsoRankN and MI-GRAAL are computationally more complex than these two algorithms.

**Table 5:** Total CPU time (min) for aligning the networks.

	DMC			DMR			CG		
	pairwise	5-way	8-way	pairwise	5-way	8-way	pairwise	5-way	8-way
IsoRank	2.5	N/A	N/A	2.5	N/A	N/A	5	N/A	N/A
IsoRankN	25	65	60	20	65	57	56	170	150
NetworkBLAST-M	0.5	10	6	0.5	10	6	0.5	10	6
Græmlin	0.3	5.5	7	0.2	3.5	7.5	0.5	5	10
MI-GRAAL	45	N/A	N/A	45	N/A	N/A	45	N/A	N/A

### Discussion

Absence of a comprehensive and reliable network alignment benchmark has been a critical obstacle that has been hindering research progress in comparative network analysis. In this work, we addressed this problem by proposing a novel network synthesis model that can generate network families with biologically realistic properties. The proposed model allows us to effectively generate families of evolutionarily related networks, where the network family may contain any number of networks with arbitrary phylogenetic relationships. We demonstrated that the internal as well as the cross-network properties of the synthesized networks closely resemble those of real protein-protein networks. Based on the proposed model, we synthesized a number of network benchmark datasets and evaluated the performance of several representative network alignment algorithms. These experiments allow us to clearly delineate the advantages and disadvantages of the respective algorithms in contrast to other algorithms. As demonstrated throughout this paper, the proposed network synthesis model provides an effective framework for generating large-scale network benchmarks, which can be used to reliably assess the performance of current and future network alignment algorithms under various conditions and problem settings.

## Supporting Information

Figure S1 **Network properties of different organisms.** (A), (B) show the degree distributions, and (C), (D) show the clustering coefficient profiles.
(EPS)

Figure S2 **Cross-species network properties for different pairs of organisms.** (A)–(N) show how the number of potential orthologs are distributed between a given pair of networks.
(EPS)

Figure S3 **Cross-species network properties for different pairs of organisms.** (A)–(G) illustrate the sequence similarity distribution for orthologous and non-orthologous node pairs.
(EPS)
